# Impact of Anti-HER2 Therapies on Overall Survival in Patients with HER2-Positive Metastatic Breast Cancer: Focusing on Intracranial Efficacy of Emerging Treatments

**DOI:** 10.3390/cancers17213520

**Published:** 2025-10-31

**Authors:** Denise Drittone, Claudia Lucci, Luisa Esposito, Federica Mazzuca, Simona Pisegna

**Affiliations:** 1 Medical Oncology Unit, Azienda Ospedaliero-Universitaria Sant’Andrea, 00189 Rome, Italy; claudia.lucci@uniroma1.it (C.L.); luisa.esposito@uniroma1.it (L.E.); federica.mazzuca@uniroma1.it (F.M.); simona.pisegna@uniroma1.it (S.P.); 2 Oncology Unit, Department of Clinical and Molecular Medicine, Sant’Andrea University Hospital, Sapienza University of Rome, 00189 Rome, Italy; 3 Department of Experimental Medicine, Sapienza University of Rome, 00161 Rome, Italy

**Keywords:** HER2-positive metastatic breast cancer, Anti-HER2 therapy, overall survival, brain metastases, intracranial efficacy, monoclonal Antibodies (MAB), antibody–drug conjugates (ADCs), tyrosine kinase inhibitors (TKIs)

## Abstract

**Simple Summary:**

Many patients with breast cancer overproduce the protein HER2, which drives aggressive tumor growth. Therapies directed at HER2 have substantially extended survival; however, up to one-third of patients still experience spread to the brain, where management is particularly challenging. This review synthesizes evidence on the significant classes of HER2-targeted therapy—monoclonal antibodies, antibody–drug conjugates, and small-molecule inhibitors—highlighting survival outcomes and activity in the brain. We appraise results from pivotal clinical trials, delineate the most effective current options, and address unresolved questions such as optimal sequencing, integration with local treatments, and selection according to tumor characteristics. We aim to provide a clear, patient-centered overview that informs clinical practice, guides future research, and supports strategies to prevent and control brain involvement.

**Abstract:**

Therapies targeting human epidermal growth factor receptor 2 (HER2) have substantially improved overall survival in patients with HER2-positive metastatic breast cancer. Approximately 31% of these patients develop brain metastases, representing a significant therapeutic challenge. This review classifies anti-HER2 therapies into three categories: monoclonal antibodies (MABs), antibody-drug conjugates (ADCs), and tyrosine kinase inhibitors (TKIs). The mechanisms of action and clinical impacts of these agents are examined, with particular attention to intracranial efficacy. The introduction of trastuzumab increased overall survival (OS) from 20.3 to 25.1 months compared to chemotherapy alone. The addition of pertuzumab further extended survival to 57.1 months, as demonstrated in the CLEOPATRA trial. Among ADCs, T-DM1 improved OS to 29.9 months versus 25.9 months in the EMILIA trial, while T-DXd extended OS to 52.6 months in DESTINY-Breast03. T-DXd also demonstrated notable intracranial activity, achieving a 64.9% objective response rate in patients with active brain metastases. In the HER2CLIMB trial, tucatinib reduced intracranial progression by 68% and improved OS (24.7 vs. 19.2 months) in patients with active brain metastases. Recent advances have increased median OS from approximately 20 months prior to trastuzumab to over 50 months with current therapies. Future research should focus on optimizing treatment sequencing, refining biomarker-driven approaches, and developing targeted strategies for brain metastases to further improve long-term survival outcomes.

## 1. Introduction

Breast cancer is the most frequently diagnosed malignancy and the leading cause of cancer-related mortality among women worldwide, with substantial geographic variation in incidence, stage at presentation, and outcomes [1]. Clinically, breast cancer is stratified by estrogen and progesterone receptor expression and by HER2 status into three clinically relevant groups—HR+/HER2− (luminal, ~70%), HER2+ (~15–20%), and triple-negative HR−/HER2− (~15%)—which exhibit distinct biology, natural history, and therapeutic sensitivity [2].

HER2-positive breast cancer accounts for approximately 20% of all cases [3]. Per the 2018 ASCO/CAP criteria, HER2-positive breast cancer (IHC 3+ or 2+/ISH+) is eligible for established anti-HER2 therapies, whereas true HER2-0 (no staining) is not, outside trials. Tumors with HER2-low (IHC 1+ or 2+/ISH−)—and the emerging HER2-ultralow subset (IHC 0 with faint, incomplete staining in ≤10% of cells)—are clinically negative for traditional agents but may access certain HER2-directed ADCs, particularly in metastatic disease; eligibility for ultralow is evolving [4]. Both HER2-positive and triple-negative subtypes are associated with poor prognoses, characterized by early and frequent recurrences and metastasis. However, the prognosis for HER2-positive disease is more favorable than for triple-negative breast cancer, primarily due to the benefits of targeted therapies [5]. The identification of EGFR in 1978 and HER2 in 1984, along with the recognition of HER2 overexpression as a negative prognostic factor, facilitated the development of targeted therapies that have transformed breast cancer management [6]. In recent decades, significant advances in targeted therapies have markedly improved outcomes for these patients. The FDA approved trastuzumab, a humanized monoclonal antibody, for HER2-positive metastatic breast cancer in 1998. This major advance improved survival and established drug-diagnostic development [7]. Trastuzumab’s success greatly influenced cancer drug development, spurring advances in HER2-targeted therapy. Pertuzumab is an anti-HER2 antibody that binds a distinct domain of the HER2 receptor compared to trastuzumab. When combined with trastuzumab and other therapies, pertuzumab can help overcome resistance and enhance treatment efficacy. It is also associated with a favorable safety profile [8]. Small-molecule kinase inhibitors, such as lapatinib and tucatinib, target HER2 and have emerged as effective treatments for HER2-positive breast cancer. These drugs have significantly improved survival outcomes for patients with advanced disease, especially when used in combination with other therapies [9]. HER2-targeting Antibody-Drug Conjugates (ADCs) mark a significant advancement in breast cancer treatment. These therapies use antibody specificity to deliver potent cytotoxic payloads directly to cancer cells. HER2-targeting ADCs, such as Trastuzumab Emtansine (T-DM1) and Trastuzumab Deruxtecan (T-DXd), use varying mechanisms of action. They show promise in treating both HER2-positive and HER2-low breast cancer, offering improved efficacy and expanding treatment options [10]. This review provides a comprehensive update on anti-HER2-targeted therapies and their impact on overall survival. The mechanisms of action of these agents are examined, and results from recent clinical trials, including phase III studies, are analyzed with respect to progression-free and overall survival. Toxicity profiles and side effects are considered to evaluate the benefit-to-risk ratio and inform strategies for optimizing clinical use. Future research perspectives are also discussed, with emphasis on novel agents, therapeutic combinations, and intracranial activity.

Beyond receptor-directed therapies, emerging biology highlights complementary avenues relevant to HER2-positive disease. In vitro studies of traditionally consumed food plants reveal antiproliferative and pro-apoptotic mechanisms in breast cancer models, supporting hypotheses for prevention research and adjuvant strategies [11]. Concurrently, bidirectional crosstalk between cancer cells and the nervous system influences tumor growth, dissemination, and brain colonization, suggesting neural signaling as a potential therapeutic vulnerability in patients at risk for CNS involvement [12]. At the cellular level, resistance to endocrine and targeted therapies in breast cancer involves multiple molecular pathways, and the identification of predictive biomarkers and druggable targets to overcome refractory disease remains an area of active investigation and continuous evolution [13].

Despite meaningful survival gains with HER2-targeted agents, substantial inter-patient heterogeneity and the emergence of primary/acquired resistance—including drug-tolerant states—limit durability of benefit; deeper mechanistic insight is needed to refine sequencing and patient selection [14].

## 2. Anti-HER2 Therapies

### 2.1. Monoclonal Antibodies (MAB)

Trastuzumab and pertuzumab, two monoclonal antibodies, have revolutionized the treatment of HER2-positive breast cancer. Both target the HER2 receptor in distinct ways. Trastuzumab was the first monoclonal antibody developed. De et al. explored how trastuzumab works, highlighting three main mechanisms. First, it disrupts the HER2–HER3 heterodimerization that drives tumor growth in HER2-overexpressing cancers. Second, it prevents the cleavage of the HER2 extracellular domain, stopping the production of the active p95HER2 fragment associated with aggressive tumor behavior. Lastly, trastuzumab activates the immune system by inducing antibody-dependent cellular cytotoxicity (ADCC), where immune cells target and destroy HER2-positive tumor cells [15]. At present, trastuzumab is used in the neoadjuvant, adjuvant, and metastatic settings in the treatment of HER2-positive breast cancer. The H0648g trial, a pivotal phase 3 study, investigated the impact of adding trastuzumab to standard chemotherapy regimens in patients with early-stage HER2-positive breast cancer. Compared to chemotherapy alone, the addition of trastuzumab demonstrated a significant improvement in key clinical outcomes in terms of progression-free survival (PFS) (median 7.4 vs. 4.6 months; *p* < 0.001) and overall survival (OS) (median 25.1 vs. 20.3 months; *p* = 0.01) [16]. A multicenter randomized trial was conducted in 2025 that demonstrated the efficacy of trastuzumab, in combination with docetaxel, in the metastatic setting of HER2-positive breast cancer. The combination resulted in higher response rates, longer overall survival (median of 31.2 months vs. 22.7 months; *p* = 0.0325), and longer time to disease progression and treatment failure, with additional manageable toxicity [17]. A prospective observational study conducted in Germany evaluated the impact of continuing trastuzumab treatment beyond disease progression in patients with advanced or metastatic breast cancer. The findings indicated that patients who received trastuzumab beyond progression experienced improved survival outcomes compared to those who did not continue the treatment. This suggests that maintaining trastuzumab therapy, even after disease progression, may offer survival benefits for patients with advanced or metastatic breast cancer [18]. In HER2-positive hormone receptor (HR)-co-positive tumors, the TAnDEM trial demonstrated improved progression-free survival with trastuzumab plus anastrozole versus anastrozole alone (4.8 vs. 2.4 months). However, overall survival was not significantly different (Median OS was 28.5 months in the trastuzumab plus anastrozole arm and 23.9 months in the anastrozole alone arm, log-rankP = 0.325), likely due to cross-over [16]. Pertuzumab primarily works by binding to the HER2 dimerization domain, blocking HER2 from associating with other HER receptors, especially HER2/HER3 complexes driven by ligands like heregulin. While it induces ADCC, this is not essential for its action, and it does not prevent HER2 shedding [19]. The combination of trastuzumab and pertuzumab represents a significant advancement in the treatment of HER2-positive breast cancer. Their synergistic action effectively targets the HER2 receptor, improving clinical outcomes with an acceptable safety profile [20]. The CLEOPATRA trial is a phase 3, randomized, double-blind trial that compared pertuzumab plus trastuzumab and docetaxel with placebo plus trastuzumab and docetaxel in patients with HER2+ metastatic breast cancer. The median OS was 57.1 months (95% CI: 50–72) in the pertuzumab group compared with 40.8 months (95% CI: 36–48) in the placebo group, with a benefit of 16.3 months for pertuzumab. One serious adverse event that occurred in the pertuzumab treatment arm was congestive heart failure. Therefore, the final results of the phase 3 CLEOPATRA study reveal that first-line treatment of HER2+ metastatic breast cancer with pertuzumab plus trastuzumab and docetaxel offers an advantage in overall survival even in the long term (37% chance of being alive in 8 years vs. 23% with placebo plus trastuzumab and docetaxel), changing the natural history of this disease [21]. The Phase IIIb PERUSE study further evaluated the double anti-HER2 blockade with trastuzumab and pertuzumab, combined with different taxane backbones, in the first-line setting. Among 1436 treated patients, median PFS was 20.7 months, with similar efficacy across hormone receptor subgroups. Paclitaxel, used in 41% of patients, showed comparable efficacy to docetaxel and nab-paclitaxel, with median PFS of 23, 19.6, and 18 months, respectively. The overall response rates (ORRs) were 83%, 79%, and 77% with paclitaxel, docetaxel, and nab-paclitaxel, respectively [22]. Median OS reached 65.3 months, particularly favorable in hormone receptor–positive patients. These findings confirm the robustness of pertuzumab-based regimens and support paclitaxel as a valid alternative to docetaxel, offering a more favorable toxicity profile with preserved efficacy [23]. These results suggest that paclitaxel is a viable alternative to docetaxel, leading to similar PFS and ORR results with a predictable toxicity profile [24]. The PHEREXA trial evaluated the benefit of adding pertuzumab to trastuzumab and capecitabine in patients with HER2-positive metastatic breast cancer who had progressed on prior trastuzumab-based therapy [25]. While the primary endpoint of progression-free survival did not reach statistical significance, the final analysis revealed a significant improvement in overall survival for patients receiving the combination of trastuzumab and pertuzumab compared to trastuzumab alone. Although the study did not meet its primary endpoint, these findings suggest that dual HER2 inhibition may offer a survival benefit in the second-line setting for patients with HER2-positive metastatic breast cancer [26]. The PERTAIN study demonstrated that the addition of pertuzumab to therapy with trastuzumab and aromatase inhibitors improved progression-free survival in patients with HER2-positive, hormone receptor-positive breast cancer, particularly in those who had not received prior chemotherapy (median 20.6 months vs. 15.8 months). In the final analysis, no significant difference in overall survival was observed between the two treatment arms. [27]. Resistance to HER2-directed monoclonal antibodies can reflect reduced immune engagement (variable TILs, FcγR polymorphisms), epitope masking (e.g., MUC4), truncated receptors (p95HER2), and downstream PI3K/AKT activation (PIK3CA mutation, PTEN loss). These alterations blunt ADCC and HER2 signaling blockade [28,29,30,31].

### 2.2. New Anti-HER2 mAB (Monoclonal Antibody)

Margetuximab is an Fc-engineered anti-HER2 immunoglobulin G monoclonal antibody that targets the same epitope as trastuzumab, with similar antiproliferative effects but a higher binding affinity for the receptor [32,33]. The SOPHIA trial showed no significant overall survival (OS) difference between margetuximab and trastuzumab in HER2-positive advanced breast cancer (21.6 vs. 21.9 months; HR 0.95); safety profiles were similar [34]. Exploratory analysis suggested OS benefits for margetuximab in CD16A-158FF patients and trastuzumab in CD16A-158VV patients. Safety profiles were similar; further studies on CD16A allelic variants are needed, like the MARGOT trial. Margetuximab is approved in the USA for HER2-positive MBC after two or more prior anti-HER2 therapies, based on SOPHIA trial data [35].

### 2.3. Antibody-Drug Conjugate (ADCs)

#### 2.3.1. Old Generation ADCs

T-DM1 is an antibody-drug conjugate (ADC) that targets the HER2 receptor. It is composed of trastuzumab, a monoclonal antibody, coupled with a cytotoxic molecule called DM1. T-DM1 acts by binding to the HER2 receptor, resulting in the internalization of the HER2-T-DM1 complex within the cell. Stable binding ensures that DM1 is released only when the antibody is degraded in the lysosome. Once released, DM1 metabolites inhibit microtubule assembly, leading to cell death. T-DM1 combines the antitumor action of trastuzumab with the cytotoxic activity of DM1 metabolites within cells [36]. Some key clinical trials have demonstrated the efficacy of T-DM1 in overall survival. The phase 3 EMILIA trial showed that T-DM1 significantly improved progression-free survival and overall survival compared with lapatinib plus capecitabine in patients with HER2-positive metastatic breast cancer who had progressed on previous trastuzumab-based therapy (29-9 months [95% CI 26-3-34-1] vs. 25-9 months [95% CI 22-7-28-3]; hazard ratio 0–75 [95% CI 0-64-0-88]) [37]. The phase 3 TH3RESA trial compared the efficacy of T-DM1 with the physician’s choice of treatment in patients with HER2-positive advanced breast cancer previously treated and with progression after at least two HER2-directed regimens. Median overall survival was significantly longer with trastuzumab emtansine (22.7 months) compared to the physician’s choice (15.8 months), with a hazard ratio of 0.68 (*p* = 0.0007) [38]. The final results confirm that trastuzumab emtansine significantly improves overall survival in these patients.

#### 2.3.2. New Generation ADCs

Trastuzumab deruxtecan (T-DXd) is a HER2-targeted antibody-drug conjugate consisting of a humanized IgG1 monoclonal antibody against HER2, a cleavable tetrapeptide-based linker, and a highly potent topoisomerase I inhibitor as its cytotoxic payload [39]. T-DXd is an internationally approved, guideline-recommended treatment for patients with HER2-positive metastatic breast cancer following progression on taxane and trastuzumab or recurrence within six months of completing neoadjuvant and/or adjuvant therapy. The most common adverse reactions (≥20%), including laboratory abnormalities, were gastrointestinal events (nausea, vomiting, constipation, diarrhea), hematologic decreases (white blood cells, neutrophils, hemoglobin, lymphocytes, platelets), elevations in hepatic parameters (AST, ALT, alkaline phosphatase, bilirubin), fatigue, alopecia, hypokalemia, musculoskeletal pain, decreased appetite, headache, respiratory infection, abdominal pain, and stomatitis. Interstitial lung disease (ILD)/pneumonitis occurred in 11% (28/257) of patients; 0.8% were grade 3, and no grade 4 or 5 ILD/pneumonitis events were adjudicated as drug-related [40]. The phase 2 DESTINY-Breast01 study evaluated the significant antitumor activity of T-DXd in patients with HER2-positive metastatic breast cancer previously treated with T-DM1 [41]. The DESTINY-Breast02 trial evaluated T-DXd versus the physician’s choice treatment (trastuzumab/capecitabine or lapatinib/capecitabine) in patients with unresectable or metastatic HER2-positive breast cancer previously treated with T-DM1. T-DXd significantly improved median PFS and showed superior efficacy with a manageable safety profile. The median overall survival, a key secondary endpoint, was 39.2 months with trastuzumab deruxtecan compared to 26.5 months with the physician’s choice of treatment [42].

These studies demonstrated substantial and durable antitumor efficacy, laying the foundation for the pivotal phase 3 DESTINY-Breast03 (DB03) trial.

The approval of T-DXd in this setting was based on the results of the DB03 trial, designed to evaluate the efficacy and safety of T-DXd compared to T-DM1. Based on the outcomes of this study, T-DXd has replaced T-DM1 as the preferred treatment in this setting [43]. At the data cutoff of November 20, 2023, the DB03 study demonstrated that T-DXd improved mOS (52.6 vs. 42.7 months; HR 0.73, 95% CI 0.56–0.94) [44]. T-DXd has shown significant overall survival benefits in HER2-positive metastatic breast cancer.

T-DXd has extended its clinical impact beyond HER2-positive disease through the phase 3 DESTINY-Breast04 trial, which operationalized the “HER2-low” category (IHC 1+ or IHC 2+/ISH-negative). Among 557 patients with unresectable/metastatic HER2-low breast cancer randomized 2:1 to T-DXd versus physician ’s-choice chemotherapy, T-DXd significantly improved outcomes. In the HR-positive primary cohort, median PFS was 10.1 vs. 5.4 months (HR 0.51) and OS 23.9 vs. 17.5 months (HR 0.64); across all randomized patients, PFS was 9.9 vs. 5.1 months and OS 23.4 vs. 16.8 months. Interstitial lung disease/pneumonitis (ILD) occurred in approximately 12% of patients, with rare fatal events, underscoring the need for early recognition and management [45].

Earlier use was subsequently evaluated in DESTINY-Breast06, which enrolled HR-positive, chemotherapy-naïve metastatic patients with HER2-low or HER2-ultralow tumors (the latter typically defined in-protocol as IHC 0 with faint/incomplete membrane staining) after ≥1 line of endocrine therapy. T-DXd significantly prolonged PFS versus chemotherapy in the HER2-low primary population (13.2 vs. 8.1 months; HR 0.62), with consistent results in the exploratory ultralow subgroup; OS data were immature at the time of the primary analysis. Adjudicated ILD occurred in ~11%, with very rare fatal events [46].

Qualifying patients with HER2-low disease for trial eligibility. Per the ASCO/CAP 2023 update, HER2-low is defined as IHC 1+ or IHC 2+/ISH-negative; it is not a new “positive” category but may be reported to facilitate therapeutic selection with T-DXd. Pathology reports should include the semiquantitative IHC score, and whenever feasible the most recent tumor specimen (archival or new biopsy) should be tested to account for heterogeneity and phenotype evolution; many pivotal protocols mandated central confirmation of HER2-low status prior to randomization [47]. When allowed by the protocol (e.g., in DESTINY-Breast06), HER2-ultralow is generally described as IHC 0 with minimal/faint membrane staining in a small fraction of cells; this construct is protocol-specific and not part of standard ASCO/CAP interpretive categories [46].

From an eligibility standpoint, line-of-therapy requirements should be respected: DESTINY-Breast04 required 1–2 prior lines of chemotherapy in the metastatic setting (with HR-positive patients endocrine-refractory), whereas DESTINY-Breast06 included patients without prior metastatic chemotherapy but with progression after ≥1 line of endocrine-based therapy; in both trials, the control arm was physician’s-choice chemotherapy. Given the non-negligible incidence of ILD, protocols emphasize patient education, a low threshold for imaging, prompt corticosteroids, and treatment interruption or discontinuation according to severity [45,46].

### 2.4. Tyrosine Kinase Inhibitor (TKI)

Tyrosine kinase inhibitors (TKIs) are small molecules that inhibit HER2 kinase activity by competing with ATP at the intracellular domain, thereby blocking PI3K/AKT and MAPK signaling that drive tumor growth and survival. Their small size and lipophilicity facilitate blood–brain barrier penetration and support efficacy against CNS metastases [48]. Despite the clinical success of monoclonal antibodies, resistance is common—e.g., truncated HER2 variants (p95HER2) lacking the extracellular domain—prompting the development of first-generation TKIs to directly inhibit the intracellular kinase domain, including truncated forms inaccessible to antibodies; several agents also inhibit multiple HER family receptors, broadening antitumor activity [31]. In HER2-positive cancers, amplification of the ERBB2 locus leads to marked overexpression of the HER2 receptor, driving constitutive activation of downstream cascades (PI3K/AKT and MAPK) that promote proliferation, survival, and apoptosis resistance. Under treatment with first-generation TKIs, high-level amplification may surpass the inhibitory capacity of these drugs, allowing persistent HER2 signaling and reducing therapeutic efficacy—thereby contributing to intrinsic or acquired resistance in HER2-amplified tumors [49,50]. An emerging mechanism of resistance to TKIs in HER2-positive tumors involves the sequestration of therapeutic agents within the tumor microenvironment by extracellular molecules secreted by cancer cells. These include components of the extracellular matrix (ECM), exosomes, and various soluble binding proteins, which can interact with TKIs and prevent their efficient diffusion and cellular uptake. Such sequestration limits the bioavailability of the drug at the tumor cell surface, effectively reducing the concentration of TKI that reaches and engages the HER2 receptor. This extracellular trapping of TKIs forms a physical and biochemical barrier to drug delivery, thereby impairing the intended inhibitory effect and contributing to therapeutic resistance [51,52].

#### 2.4.1. Old Generation TKI

Lapatinib is a small-molecule tyrosine kinase inhibitor that targets EGFR and HER2, with unclear effects on pyruvate kinase type M2 (PKM2), and is used in the treatment of advanced HER-2-positive breast cancers [53,54]. In 2017, a systematic review analyzed 12 studies involving 799 patients with HER2-positive breast cancer and brain metastases (BMs) treated with lapatinib (L), alone or with capecitabine (C). The pooled overall response rate (ORR) was 21.4% (95% CI 11.7–35.9) and increased to 29.2% (95% CI 18.5–42.7) for L + C. The pooled median progression-free survival (PFS) was 4.1 months (95% CI 3.1–6.7), and overall survival (OS) was 11.2 months (95% CI 8.9–14.1) [55]. The phase II VITAL study found that lapatinib plus vinorelbine (lap + vin) had comparable efficacy and safety to lap + cap, with a median OS of 23.3 vs. 20.3 months and similar death rates (56–57%), supporting lap + vin as a treatment option for HER2-positive MBC [56].

The emergence of resistance to first-generation tyrosine kinase inhibitors (TKIs) has been a significant limitation in the long-term management of cancers driven by aberrant tyrosine kinase signaling, including HER2-positive breast cancer. First-generation TKIs, such as lapatinib, typically act through reversible binding to the ATP-binding site of the kinase domain. However, this binding is often insufficient to achieve durable target suppression, particularly in the presence of acquired resistance mutations, which increase ATP affinity or directly impair drug binding [57,58].

Furthermore, HER2-driven signaling often involves heterodimerization with other members of the ErbB family (HER1/EGFR, HER3, HER4), enabling signal propagation even in the presence of HER2 blockade. Consequently, there is a clinical rationale for the development of second-generation TKIs capable of broader and more durable inhibition across multiple ErbB family members [59,60]. Resistance to first-generation HER2 TKIs, such as lapatinib, arises from several mechanisms that limit long-term therapeutic efficacy in HER2-positive breast cancer. Activation of the downstream PI3K/AKT signaling, often through PIK3CA mutations or PTEN loss, enables HER2-independent cell survival [57,58]. Acquired HER2 mutations (e.g., T862A, L755S) reduce TKI binding and sustain signaling, while truncated forms like p95HER2 evade antibody therapies and alter drug sensitivity. Resistance is also mediated by anti-apoptotic proteins (e.g., MCL-1, XIAP) and impaired pro-apoptotic signaling (e.g., FOXO3a, c-FLIP) [31,61]. These mechanisms often coexist within tumors, leading to complex, multi-layered resistance that necessitates combination strategies. Approaches such as dual inhibition of HER2 and downstream effectors (e.g., PI3K, AKT, MEK), as well as the use of irreversible pan-HER TKIs (e.g., neratinib), are being explored to overcome resistance [62].

Neratinib is a pan-HER tyrosine kinase inhibitor that blocks HER signaling, induces cell cycle arrest, downregulates HER2, and reverses multidrug resistance [63]. The phase III NALA trial demonstrated that neratinib plus capecitabine (N + C) significantly improved progression-free survival (PFS) and reduced central nervous system (CNS) disease; however, overall survival (OS) was not significantly different (HR 0.88; *p* = 0.2098) compared to lapatinib plus capecitabine (L + C) in patients with HER2-positive metastatic breast cancer [64].

#### 2.4.2. New Generation TKI

Tucatinib is a selective and reversible inhibitor of HER2 protein tyrosine kinase activity, with minimal impact on EGFR inhibition, that has become a valuable treatment option for patients with HER2-positive MBC who have progressed on prior therapies [65]. The pivotal HER2CLIMB trial evaluated the efficacy and safety of tucatinib combined with trastuzumab and capecitabine in patients with HER2-positive metastatic breast cancer, including those with brain metastases—a population historically excluded from clinical trials. Final analysis showed that the tucatinib combination significantly improved overall survival (OS) compared to the placebo combination, with a median OS of 24.7 months versus 19.2 months (hazard ratio [HR]: 0.73, *p* = 0.004) and a 2-year OS rate of 51% versus 40%. Progression-free survival (PFS) was also notably extended [66]. The study confirmed that tucatinib, when combined with trastuzumab and capecitabine, provides a clinically meaningful survival benefit for patients with HER2-positive metastatic breast cancer. An exploratory analysis of HER2CLIMB data focusing on patients who experienced isolated CNS progression and continued on study-assigned treatment after local intervention demonstrated a prolonged median time to second progression (either intracranial or systemic) of 15.9 months in the tucatinib arm versus 9.7 months in the control group (HR: 0.29) [67]. Overall, HER2CLIMB represents the first randomized clinical trial to demonstrate an OS benefit in patients with active brain metastases from HER2-positive breast cancer. These results firmly support the therapeutic positioning of tucatinib, in combination with trastuzumab and capecitabine, as an effective option for patients with CNS-involved disease [68].

#### 2.4.3. Non-HER2 Tyrosine Kinase Inhibitors in Combination with Anti-HER2 Therapies

In a phase II, open-label, single-arm clinical trial, Bachelot et al. showed that the combination of sunitinib and trastuzumab had modest antitumor activity in HER2-positive advanced breast cancer, with greater benefit in less heavily pretreated patients; however, cardiac toxicity and other adverse events remain concerns, and the overall benefit did not appear superior to established dual HER2-targeted regimens [69].

For dasatinib (a Src/Abl TKI), single-agent activity in advanced HER2-positive and/or HR-positive breast cancer was limited in a phase II study (short treatment duration and low disease-control rate) [70]. By contrast, the combination of dasatinib + trastuzumab + weekly paclitaxel as first-line therapy in the GEICAM/2010-04 phase II trial yielded a high objective response rate (~79%) and a median PFS around 24 months, with manageable toxicity; being single-arm, these results require randomized confirmation [71,72]. Regarding imatinib + trastuzumab, an early phase I dose-escalation trial across HER2/neu-overexpressing tumors explored feasibility and safety, without establishing efficacy or a role in routine HER2-positive breast cancer treatment [73].

### 2.5. Intracranial Efficacy of Anti-HER2 Therapies

Brain metastases (BMs) represent the most lethal subset of metastatic breast cancer, highlighting the need for greater focus after diagnosis and underscoring the importance of developing individualized management strategies for these high-risk patients [74]. The incidence of BMs among patients with HER2-positive breast cancer was 31% [75]. HER2-positive patients had the longest median survival after CNS metastases (16 months), and HER2 positivity emerged as an independent prognostic factor for improved outcomes (HR 0.60, *p* = 0.007) [76]. Local therapies help control breast cancer brain metastases, while systemic treatments, including chemotherapy and targeted agents, are increasingly essential due to the cognitive risks of whole-brain radiotherapy [77]. Surgery is recommended for single BMs and may be considered for multiple resectable BMs; stereotactic radiosurgery (SRS) is the preferred option for 1–4 BMs and may be considered for 5–10 BMs with limited tumor volume. Whole-brain radiotherapy (WBRT) should be avoided after surgery or SRS but may be considered for multiple unresectable BMs when CNS-active systemic therapy is unavailable [78]. Highlighted by Fontanella et al., therapeutic progress in this area has long lagged behind extracranial disease management, leaving patients with brain metastases underrepresented in clinical trials and often without effective systemic options [79]. This unmet need has gradually prompted the development of CNS-active anti-HER2 agents and the inclusion of patients with brain metastases in pivotal studies, reshaping the current treatment paradigm. Two retrospective studies evaluated the efficacy of first-line trastuzumab plus pertuzumab (TP) and a taxane in HER2-positive mBC with BMs. The Reper study (n = 21) reported an ORR of 52.4% and a median PFS of 20 months, while the Bergen et al. study (n = 26) showed a higher ORR of 92.9%, but with a shorter PFS of 8 months and a median OS of 44 months [80,81]. However, both studies had a high risk of bias due to their retrospective design and the use of local therapies. The phase II PATRICIA study assessed high-dose trastuzumab (HDT) plus pertuzumab after progression on standard trastuzumab in pretreated patients (n = 39), showing a low CNS-ORR of 11% but confirming the safety of HDT [82]. The EMILIA trial T-DM1 demonstrated a significant overall survival advantage (26.8 vs. 12.9 months; HR = 0.38; *p* = 0.008) over capecitabine–lapatinib (XL), despite similar progression-free survival between the groups. The incidence of CNS progression was higher among those with baseline brain metastases but remained comparable between treatments [83]. The objective-response rate was higher in the T-DM1 group (43.6%; 95% CI, 38.6 to 48.6) than in the lapatinib–capecitabine group (30.8%; 95% CI, 26.3 to 35.7; *p* < 0.001), and the median duration of response was longer (12.6 months vs. 6.5 months). The KAMILLA phase IIIb study confirmed T-DM1′s activity in HER2-positive MBC with BMs, supporting further research. Notably, in patients with previously untreated, asymptomatic BMs, T-DM1 achieved an intracranial objective response rate (ORR-IC) of 49.3%, highlighting its potential efficacy in this subgroup [84]. A pooled analysis of the DEBBRAH, TUXEDO-1, and DFCI/Duke/MDACC studies confirmed the significant intracranial efficacy of T-DXd in HER2-positive breast cancer with active brain metastases, achieving an ORR-IC of 64.9% and a clinical benefit rate of 81.1%. T-DXd maintained a stable quality of life and presented no new safety concerns, supporting its use regardless of brain metastasis status [85]. The DESTINY-Breast12 study confirmed T-DXd as a highly effective treatment for HER2-positive breast cancer, with BMs overcoming the blood–brain barrier, achieving a CNS-specific PFS of 58.9% and an ORR of 71.7%, which significantly improved patient outcomes [86]. The HER2CLIMB study showed that adding tucatinib to trastuzumab and capecitabine significantly improved outcomes in HER2-positive breast cancer with BMs, reducing intracranial progression or death by 68% and doubling the intracranial objective response rate (47.3% vs. 20.0%), establishing its role as an effective treatment option [87].

**Figure 1:**
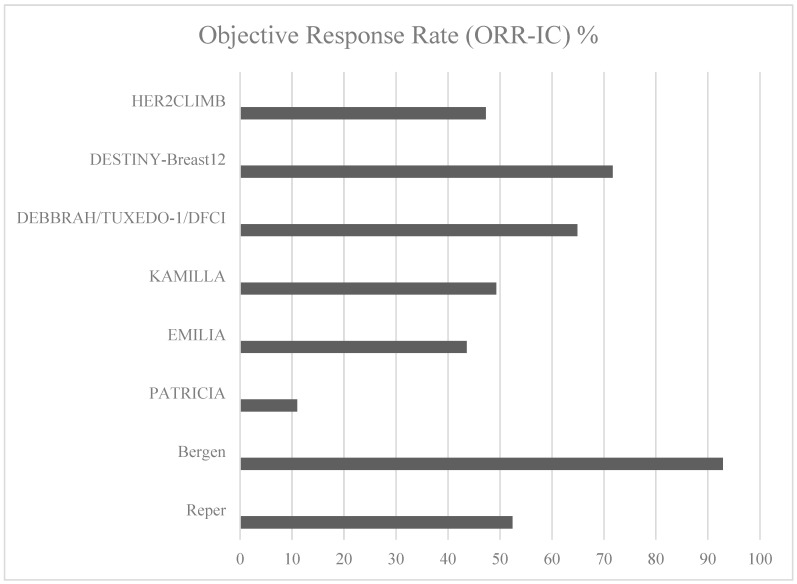
Median overall survival with Anti-HER2 therapies.

The intracranial efficacy of anti-HER2 therapies has improved substantially over time, as demonstrated by increasing objective response rates (ORR-IC) in recent studies such as HER2CLIMB and DESTINY-Breast12 (see Figure 1). These findings highlight significant advancements in systemic therapies for HER2-positive breast cancer with brain metastases. The expanding role of targeted treatments has improved survival and quality of life, yet further research is needed to refine therapeutic strategies for this high-risk population.

## 3. Discussion

The evolution of anti-HER2 therapies has dramatically transformed the treatment landscape for HER2-positive breast cancer, with successive generations of treatments demonstrating increasingly impressive survival benefits. This progression highlights both the remarkable advances in targeted therapy and areas requiring further investigation. The introduction of trastuzumab marked the first significant improvement in overall survival (OS), extending median OS from 20.3 to 25.1 months compared to chemotherapy alone. The addition of pertuzumab to trastuzumab and docetaxel (CLEOPATRA trial) further elevated the survival benchmark, achieving a median OS of 57.1 months compared to 40.8 months with trastuzumab and docetaxel. This 16.3-month improvement represented a paradigm shift in HER2-positive metastatic breast cancer treatment, with 37% of patients surviving at 8 years versus 23% in the control arm. The development of antibody-drug conjugates (ADCs) has further extended survival benefits. T-DM1 demonstrated superior efficacy compared to lapatinib plus capecitabine, with a median OS of 29.9 months versus 25.9 months in the EMILIA trial. More recently, trastuzumab deruxtecan (T-DXd) has shown remarkable results in the DESTINY-Breast03 trial, achieving a median OS of 52.6 months compared to 42.7 months with T-DM1. This evolution from trastuzumab to T-DXd reflects a steady and meaningful improvement in OS, now exceeding 50 months with optimal sequencing. While survival has improved significantly, challenges remain—particularly in patients with brain metastases. From 1998 to 2024, each new therapeutic approach has contributed to extending patient survival, with the most recent data showing an unprecedented duration of survival for metastatic disease. This advancement demonstrates not only the success of targeted therapy development but also the importance of continued innovation in the field.

Notwithstanding these gains, HER2-positive tumors represent only ~11–15% of breast cancers in the United States, so the remarkable progress with anti-HER2 agents benefits a biologic subset of patients [88,89]. By contrast, triple-negative breast cancer (TNBC)—~10–15% of cases—lacks HER2 and hormone-receptor expression and remains clinically challenging with fewer validated targets; although recent advances such as pembrolizumab in PD-L1–positive disease and the TROP2-directed antibody–drug conjugate sacituzumab govitecan have improved outcomes, overall survival remains inferior to that seen in HER2-positive disease, underscoring an ongoing unmet need [90,91].

A critical advancement in anti-HER2 therapy has been the increasing focus on intracranial efficacy. Brain metastases, affecting approximately 31% of HER2-positive breast cancer patients, have historically represented a significant therapeutic challenge. Recent developments, particularly with newer agents, have shown promising intracranial activity. T-DXd has demonstrated remarkable efficacy in patients with brain metastases, achieving an intracranial objective response rate of 64.9% and maintaining quality of life. Similarly, the addition of tucatinib to trastuzumab and capecitabine reduced intracranial progression or death by 68%, representing a significant advance in managing this challenging patient population. These findings underscore the critical importance of including patients with brain metastases in clinical trials. Historically, these patients have often been excluded from pivotal studies, which has limited our understanding of treatment efficacy in this high-risk population. The success of newer agents in treating brain metastases underscores the importance of systematically including these patients in future clinical trials to enhance understanding of therapeutic options and optimize treatment strategies. Beyond intracranial control, a deeper understanding of tumor biology may unlock personalized strategies.

Heterogeneity of HER2 in metastatic lesions is a key driver of non-response [4,45,47]. Spatial and temporal variation in antigen density (positive–low–ultralow) and clonal evolution under therapy can reduce antibody binding and ADC delivery, while ERBB2 kinase-domain mutations and p95HER2 variants, together with PI3K/AKT reactivation (PIK3CA mutation, PTEN loss), sustain signaling despite HER2 blockade [14,28,31,61]. Host and microenvironmental factors—including variable TILs and FcγR polymorphisms, as well as stromal/exosome-mediated sequestration and the blood–brain barrier—further blunt efficacy, particularly in the CNS [28,29,51,52,77]. Routine re-biopsy of progressing sites and/or ctDNA profiling should be used to reassess HER2 status and co-alterations, enabling biomarker-guided sequencing and rational combinations [47,92].

**Figure 2:**
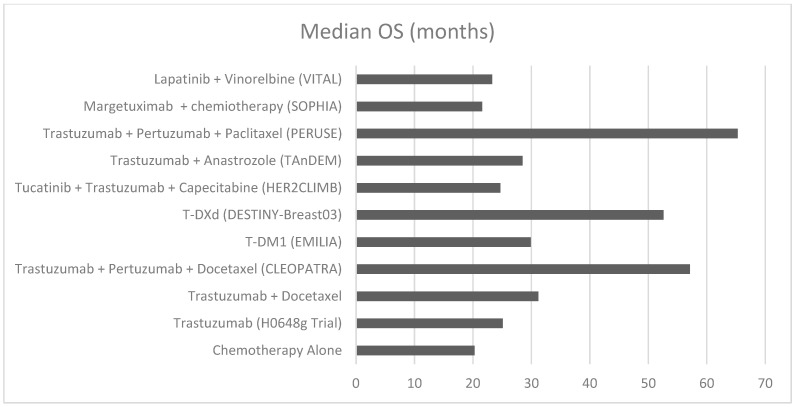
Intracranial objective response rates (ORR-IC) in HER2-positive breast cancer.

Despite these advances, predictive biomarkers remain crucial to optimizing treatment selection and outcomes. The recognition of CD16A polymorphisms has emerged as a potential predictor of response to trastuzumab-based therapies, as seen in the SOPHIA trial with margetuximab. Refining patient selection through biomarker integration is the next frontier in personalizing HER2-positive mBC therapy. Expanding on this, the optimization of treatment selection based on tumor biology is essential for achieving durable responses in HER2-positive metastatic breast cancer. Individualized management strategies should incorporate HER2 heterogeneity, hormone receptor co-expression, ERBB2 mutations, and features of the tumor microenvironment. These factors influence treatment sensitivity and resistance and can guide the sequencing of anti-HER2 agents. Integrating molecular profiling and multidisciplinary decision-making will be key to personalizing care and improving long-term outcomes. The GIM14 BIO-META study highlighted that HER2 IHC score 3+, non-visceral metastases, and a single metastatic site are associated with radiologic complete response (rCR) to first-line anti-HER2 therapy. Among long responders (TTD >18 months), median OS reached 12.7 years, with a 5-year survival rate of 92%, supporting the potential for treatment de-escalation in selected patients. Liquid biopsy and MRD assessment may further refine this approach, as explored in the STOP-HER2 trial [92]. Future research should focus on validating these biomarkers to enable more personalized treatment strategies. Additionally, real-world evidence (RWE) is crucial for understanding how clinical trial results are applied in routine clinical practice. Retrospective analyses and large observational studies can provide insights into treatment effectiveness across diverse patient populations, including those typically underrepresented in trials, such as elderly patients and those with brain metastases. Integration of RWE into treatment guidelines could further refine therapeutic decision-making. The availability of multiple effective anti-HER2 therapies underscores the critical importance of developing optimal treatment sequencing strategies, as the choice and order of therapies can significantly impact patient outcomes. A well-planned sequential approach, considering factors such as prior treatments, resistance patterns, and the presence of brain metastases, is fundamental for maximizing survival benefits and maintaining quality of life. Looking forward, several key areas require attention. First, while survival improvements have been substantial, there remains a need for biomarker-driven approaches to better predict treatment response and resistance. Second, the optimal sequencing of available therapies needs further investigation, particularly given the expanding therapeutic arsenal. Finally, the development of strategies to prevent or delay the onset of brain metastases represents an important area for future research. The remarkable progress in anti-HER2 therapy exemplifies the success of targeted approaches in oncology. A longitudinal comparison of median overall survival (OS) across pivotal clinical trials demonstrates the steady improvement in outcomes with successive generations of anti-HER2 therapies (Figure 2). These findings highlight the importance of translating clinical trial results into routine clinical practice. Observational data from the GIM14/BIOMETA study support this need by documenting a progressive improvement in overall survival for patients with HER2-positive metastatic breast cancer over time, in parallel with the introduction of novel anti-HER2 therapies and evolving treatment strategies in real-world settings. Bridging the gap between clinical trials and real-world implementation remains crucial to translate the benefits of anti-HER2 therapies into tangible outcomes for diverse patient populations. However, continued research efforts are essential to further improve outcomes, particularly for patients with brain metastases. Future clinical trials should prioritize the inclusion of these high-risk patients to ensure that emerging therapies are effective across the full spectrum of disease presentations.

## 4. Conclusions

The development of anti-HER2 therapies has markedly improved overall survival in HER2-positive metastatic breast cancer, increasing median survival from 20 months to over 50 months with current treatments. Recent innovations, such as T-DXd (DESTINY-Breast03) and tucatinib-based regimens (HER2CLIMB), have further enhanced survival, particularly for patients with brain metastases. However, challenges persist regarding optimal therapy sequencing, resistance mechanisms, and early intervention strategies. As the therapeutic landscape evolves, effectively translating clinical trial findings into routine practice and addressing resistance will be critical for further improving patient outcomes and advancing personalized treatment approaches. In future updates, we will expand our analysis of resistance and tolerance mechanisms—clarifying why HER2-targeted therapies yield divergent outcomes across patients—to inform biomarker-driven selection and next-generation combination strategies. 

In future practice, treatment selection and sequencing should be individualized based on patient- and tumor-specific factors. Key determinants include tumor biology (HER2 expression across the positive–low spectrum, intratumoral heterogeneity, hormone-receptor coexpression, ERBB2/PIK3CA alterations), disease pattern and tempo (especially CNS involvement), and host features (immune milieu/TILs, performance status, comorbidities, prior toxicities such as ILD risk with T-DXd). Longitudinal biomarkers—rebiopsy and circulating tumor DNA/Minimal Residual Disease—should be used to detect resistance mechanisms and adapt therapy (including rational combinations targeting PI3K/AKT/MAPK). Embedding these variables into multidisciplinary, patient-centered algorithms can operationalize truly personalized care. Ongoing integration of molecular diagnostics, real-world evidence, and patient-focused trial design will be essential to fully realize the potential of anti-HER2 therapy in metastatic breast cancer.

## Data Availability

No new data were created or analyzed in this study. Data sharing is not applicable to this article.
